# Early Treatment of Ruptured Blood Blister-Like Aneurysms of the Internal Carotid Artery With Flow Diverters Using Single Antiplatelet Therapy: A Single-Center Experience With Long-Term Follow-Up

**DOI:** 10.3389/fneur.2021.708411

**Published:** 2021-09-22

**Authors:** Anil Tanburoglu, Cagatay Andic

**Affiliations:** ^1^Neurology Department, Başkent University, Adana, Turkey; ^2^Radiology Department, Başkent University, Adana, Turkey

**Keywords:** blood blister-like aneurysms (BBAs), flow diverter, single antiplatelet therapy, early treatment, ruptured, P2Y12 antagonists, prasugrel, ticagrelor

## Abstract

**Background and Purpose:** Blood Blister-like aneurysms (BBAs) of the internal carotid artery (ICA) are rare entities of cerebral aneurysms. FD use in acutely ruptured aneurysms, timing of treatment and antiplatelet regimen are main debate topics in clinical practice when the treatment decision is flow diversion. The aim of this study is to report the safety and efficacy of a single-center FD treatment for ruptured BBAs in the early phase of SAH using the SAPT regimen.

**Material and Method:** This study involved a retrospective analysis of a prospectively collected database. Records of patients admitted to our clinic and treated by endovascular route on ruptured BBA between January 2013 and December 2020 were reviewed. Ruptured supraclinoid ICA BBAs treated with FD devices with SAPT within 48 h from ictus of SAH are included. BBA of atypical anatomic locations, other endovascular techniques performed, and delayed admissions (>48 h) were excluded from the study. Demographic, clinical and angiographic features of patients and aneurysms, FD types and numbers, periprocedural complications, immediate and follow-up angiographic and clinical outcomes were recorded.

**Results:** A total of six patients with ruptured BBAs treated via FDs within 48 h and used SAPT were included in the study. The mean age was 41.6 years (range from 34 to 45 years), and four of six patients were female. All patients were treated within 48 h after ictus, and the mean treatment day was 1.33 days. One patient received ticagrelor, and five patients received prasugrel as SAPT for one year after treatment. No procedure-related death and rebleeding were recorded. One (16.7 %) treatment responsive procedure-related complication occurred (transient ischemia). Overall good outcome rate was 83.3%. One patient died due to pneumonia. The immediate control angiograms showed complete occlusions of BBAs in one patient (16.6%). The complete occlusion rate was 100 % for five survivors at the control angiogram. The median follow-up was 49.5 months.

**Conclusion:** This single-center experience suggests that early treatment (<2 days) within SAH of ruptured BBAs with FDs using SAPT is safe and effective in terms of clinical and radiological long-term outcomes.

## Introduction

Blood blister-like aneurysms (BBAs) of the internal carotid artery (ICA) are a rare entity of cerebral aneurysms with extremely thin, fragile walls and a wide, poorly defined neck, originating at non-branching segments of the supraclinoid ICA (1). They are attributed to sub-adventitial dissections leading to a focal wall defect with the absence of internal elastic lamina and media, usually resulting in acute subarachnoid hemorrhage (SAH) (2). They account for 1% of all intracranial aneurysms and 0.5–2% of all ruptured aneurysms (3). BBAs are prone to rerupture; therefore, urgent diagnosis and treatment are mandated (4). BBAs have proven difficult to manage surgically or endovascularly due to their fragile state and difficult morphology (5). There has been emerging evidence favoring endovascular treatment options and advances in device technology, focusing on reconstructive treatment, especially *via* flow diversion (6). Although there have been reports of BBA treatment with flow diverter (FD) devices with promising results, FD use in acutely ruptured aneurysms is still under debate due to the risk of rerupture, delayed occlusion, and the need for antiplatelet therapy in the acute setting of SAH (7).

Timing of treatment and antiplatelet regimen are two main debate topics in clinical practice when the treatment decision is flow diversion. Flow diversion strategy for ruptured intracranial BBAs in a delayed fashion has been evaluated in the literature. Still, there are limited data about the safety, feasibility, and long-term outcomes of early management (<2 days) of BBAs with FDs (7–9). Dual antiplatelet therapy (DAPT) is commonly used to prevent thromboembolic events for FD treatments. However, hemorrhagic complications are a major concern during DAPT treatment in the acute setting of SAH. There have been limited data obtained from previous studies on single antiplatelet therapy (SAPT) in preventing thromboembolic complications associated with FDs (10). New-generation P2Y12 antagonist (ticagrelor and prasugrel) usage in neurointervention is a novel topic (11). This study aims to report the safety and efficacy of a single-center FD treatment for ruptured BBAs in the early phase of SAH using new-generation P2Y12 antagonists as SAPT regimen.

## Materials and Methods

This study involved retrospective analysis of a prospectively collected database after approval from the institutional review board. Records of patients admitted to our clinic and treated by endovascular route on ruptured BBA between January 2013 and December 2020 were reviewed. Digital subtraction angiography (DSA) was used to diagnose SAH due to BBAs. BBAs were defined according to Zhao et al., and due to the ongoing debate of the definition of BBAs, only non-branching ICA BBAs are defined as BBAs (1). Ruptured supraclinoid ICA BBAs treated with FD devices with SAPT within 48 h from ictus of SAH are included. Exclusion criteria were the following: (1) BBA of atypical anatomic locations (i.e., basilar artery and anterior cerebral artery) and (2) other endovascular techniques performed (i.e., stent-assisted coiling).

Data were gathered by using the following: demographic features of patients, clinical symptoms, Glasgow Coma Scale (GCS) scores, Hunt and Hess grades, Fisher computed tomography (CT) rating scales, aneurysm measurements, localization, the timing of treatment from ictus, FD types and numbers, periprocedural complications, external ventricular drainage requirement, antiplatelet loading dose and maintenance regimen, and immediate and follow-up angiographic and clinical outcomes.

### Treatment and Technique

After the diagnosis of ruptured BBA of ICA in diagnostic angiography, if external ventricular drainage (EVD) was required, treatment was performed just after EVD insertion (in one of six patients). However, if EVD is not indicated (five of six patients), treatment was performed in the first 48 h of ictus. All patients were treated under general anesthesia. Working projections, main artery diameters, and aneurysm dimensions were obtained using angiography together with three-dimensional (3D) rotational angiography. The tri-axial system was used in all cases *via* femoral access; six French (F) 80-cm-long sheath, 5–6 F intermediate, intracranial support or distal access catheter, and 0.027” microcatheter suitable for the FDs. While FD size was chosen according to the maximal diameter of the parent artery, FD device type was chosen based on availability. The FDs were then deployed across the neck of BBA under fluoroscopy in order to cover 3 mm proximal and distal to the aneurysm. Overlapping FD decision was made by the treating interventionist according to the margin diameters. After the immediate control, angiograms obtained from all patients were taken to the intensive care unit.

### Antiaggregant/Anticoagulant Protocol

All procedures were performed under systemic heparinization (initial heparin bolus of 5,000 IU, according to the targeted 250–300 s of activated clotting time, 1,000–2,500 IU additional boluses). Heparinization was discontinued after endovascular treatment. One of six patients received a ticagrelor 180-mg loading dose *via* nasogastric tube 2 h before treatment and 90 mg twice daily for 1 year. Five of six patients received prasugrel 60 mg loading dose 30 min before FD deployment *via* nasogastric tube and 10 mg for maintenance for 1 year. After the first 24 h and 7 days of treatment, thrombocyte inhibition levels were confirmed by the rapid platelet function Assay VerifyNow P2Y12 (Accumetrics, San Diego, California, USA) and an inhibition value of >30% was accepted for treatment. All levels measured were higher than >30%. After the first year control, acetylsalicylic acid treatment was continued indefinitely in all patients.

### Follow-Up

Clinical follow-up was performed at 3 months after discharge. Functional outcome was assessed using the GCS at discharge, modified Rankin Scale (mRS) at discharge, and at 3 months. The mRS ≤ 2 was considered as a good outcome and clinical safety parameter. Control DSA was performed at 3 months after treatment and repeated every 1 year for long-term follow-up. Aneurysm occlusion was evaluated according to the O'Kelly–Marotta (OKM) grading scale (12). Complete obliteration, need for retreatment, and absence of rebleeding were the efficacy parameters. Patients were followed up for at least 28 months after treatment.

## Results

### Patient Data and Aneurysm Characteristics

A total of six patients with ruptured BBAs treated *via* FDs within 48 h and used SAPT were included in the study. The mean age was 41.6 years (range from 34 to 45 years), and four of six patients were female. Of six patients, two patients were admitted with GCS score 15, two patients with 13, one patient with 7, and one patient with 5. The initial Hunt–Hess grade was I in one (16.6%) patient, II in three (50%) patients, and V in two (33.3%) patients. Fischer CT grade was I in three (50%) patients, III in two (33.3%) patients, and IV in one (16.6%) patient. EVD was placed in one patient before the treatment, and none of the patients required EVD after the treatment. Of the patients, four (66.7%) were treated in the first 24 h after the ictus, and two (33.3%) were treated in the 24- to 48-h period. The mean treatment day was 1.33 days. All BBAs were located at the supraclinoid ICA. The median aneurysm height was 1.50 mm (range from 1 to 2 mm), and the median aneurysm diameter was 3 mm (range from 2 to 4.5 mm). Overlapping FDs were performed in three cases, and a single FD was used in three cases (Table 1).

**Table 1:** Clinical and radiological characteristics of the patients.

**Case no**	**Gender**	**Age years**	**Hypertension**	**Smoking status**	**Fisher CT rating scale**	**Hunt-Hess grading**	**GCS at admission**	**EVD insertion**	**aneursym size (height-neck)**	**Dome/neck ratio**	**Time to treatment (days)**	**Stent type, stent number**
1	F	48	–	No	1	1	15	No	1–4.6 mm	<1	2	PED, 2
2	F	34	+	No	3	2	13	No	1.5–2.5 mm	<1	1	PED, 2
3	M	43	+	No	3	5	7	Yes	1.5–2 mm	<1	1	PED,1
4	F	36	–	No	1	2	13	No	2–4.5 mm	<1	1	DED,1
5	F	45	+	No	1	2	15	No	2–3mm	<1	2	PED, 1
6	M	44	+	No	4	5	5	No	1–2 mm	<1	1	SURPASS, 1+FRED,1

*No, number; F, female; M, male; CT, computed tomography; GCS, Glasgow Coma Scale; PED, Pipeline Embolization Device (ev3/Covidien/Medtronic, Irvine, CA); DED, Derivo Embolization Device (Acandis GmbH & Co. KG, Pforzheim, Germany); SURPASS, Surpass streamline stent (Stryker Neurovascular, Stryker, Kalamazoo, MI); FRED, Flow-Redirection Endoluminal Device (MicroVention, Inc.)*.

### Periprocedural Complications

One patient died 5 days after the treatment due to pneumonia. One patient developed ipsilateral vision loss that lasted 12 h due to vasospasm and was treated with intra-arterial nimodipine. No other intraoperative or treatment-related complication was observed. The procedure-related complication rate was 16.7%. No other periprocedural thromboembolic or hemorrhagic complications were observed. After the initial treatment, none of the patients needed retreatment for rebleeding (Table 2).

**Table 2:** Clinical and radiological outcomes, follow-up measures and antiplatelet regimen.

**Case no**	**Immediate angiographic outcome (OKM)**	**Periprocedural complication**	**GCS at discharge**	**mRS at discharge**	**mRS at 90 days**	**First control angiogram date**	**Control angiographic outcome (OKM)**	**In stent stenosis**	**Follow up months**	**Antiagregant**
1	D	No	15	0	0	3 months	D	–	82 months	ticagrelor
2	C	Transient vision loss	15	0	0	3 months	D	–	67 months	prasugrel
3	B	No	15	2	1	3 months	D	–	60 months	prasugrel
4	C	No	15	0	0	3 months	D	–	60 months	prasugrel
5	C	No	15	0	0	2 months	D	–	28 months	prasugrel
6	B	Death	ex	6	6	Ex	ex	–	ex	prasugrel

*No, number; OKM, O'Kelly-Marotta grading scale; GCS, Glasgow Coma Scale; mRS, modified Rankin Scale*.

### Clinical and Radiological Outcome

Four of six patients were discharged with mRS 0. One patient discharged with mRS 2 was later assessed mRS 1 at 3 months follow-up. Overall good outcome rate was 83.3. No procedure-related mortality was recorded.

**Figure 1:**
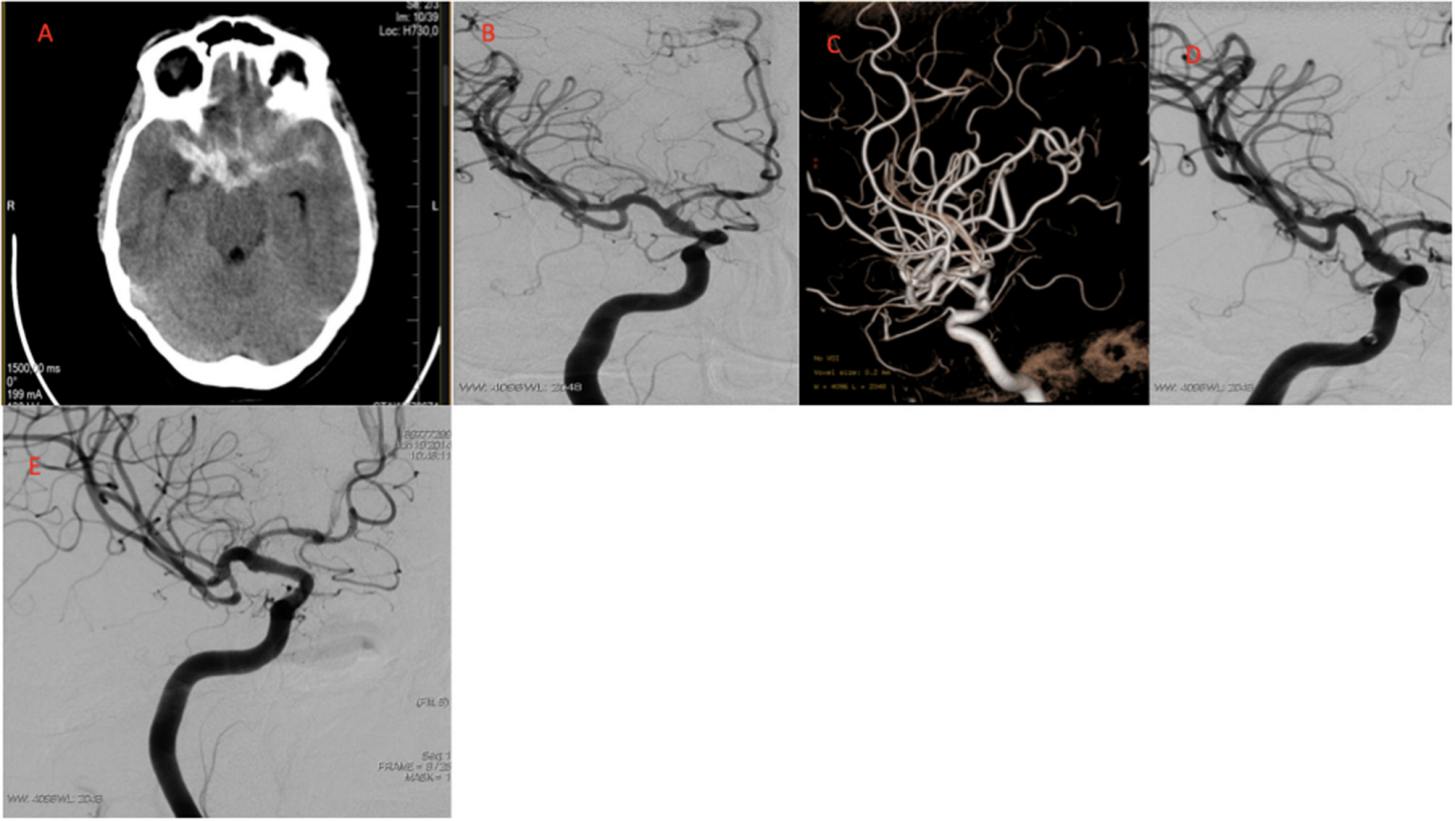
Images of a typical case. **(A)** CT images after headache showing SAH Fisher CT grade 3. **(B)** Conventional DSA obtained before treatment showing supraclinoid ICA BBA. **(C)** 3D angiogram of BBA. **(D)** Immediate control angiogram OKM C type anurysm filling. **(E)** 3 month control angiogram OKM D type complete obliteration. CT, computed tomography; SAH, subarachnoid haemorrhage; DSA, Diagnostic cerebral angiography; BBA, Blood blister like aneurysm; OKM, O'Kell–Marotta grading scale.

The immediate control angiograms showed complete occlusions of BBAs in one patient (16.6%) (OKM D). Three patients (50%) had residual neck (OKM C) and two patients (33.3%) had residual aneurysm (OKM B) on the immediate control. The immediate complete occlusion rate was 16.6%. All five surviving patients underwent DSA control at 3 months after treatment. Complete occlusion was observed in all patients at the first control angiogram (OKM D) (Figure 1). The complete occlusion rate was 100% for five patients. All five survivors from SAH treated by FDs had a long-term clinical and angiographic follow-up for at least 28 months after the procedure. The median follow-up was 49.5 months. Complete occlusion and FD patency were observed in all five patients in annual DSA controls (Table 2).

## Discussion

This study aims to report the safety and efficacy of a single-center FD treatment for ruptured BBAs in the early phase of SAH using SAPT regimen. A definitive diagnosis of BBA can be made by only histopathologic studies. Although some morphologic and radiologic features can define a BBA, the diagnostic criteria of BBAs have not been established yet. Comparison of characteristics between BBAs and non-BBAs of supraclinoid ICA suggests that the dome/neck ratio < 1 and distal angle of BBAs are two radiologic features for diagnostic accuracy (1). In this study, all aneurysms' dome/neck ratio was < 1, and this finding was consistent with the literature. Ruptured BBA patients are younger than those with saccular aneurysms, and some authors have reported female dominance and association with systemic arterial hypertension (2, 13). Four of six patients (66.7%) were female, five patients (83.3%) had systemic hypertension, and the mean age was 41.6 years. These data are in line with those observed in relevant studies (2, 13–17). The pathogenesis of BBAs remains uncertain, but recent review indicates that atherosclerosis and dissection are the main prerequisites for the formation of BBAs, and hemodynamics may play a role in the process of BBA formation due to the unique vascular anatomy of the supraclinoid ICA (18). All these mechanisms lead to extremely fragile aneurysms, a propensity to rupture at a small size, aggressive early-stage morphological progression (regrowth), and rerupture (19). In their single-center retrospective analysis of 81 patients, Wen et al. concluded that short-term progressive BBAs and non-progressive BBAs might represent different stages during BBA development rather than two distinct clinical patterns (20). The study also recommended early intervention, regardless of treatment methods, for progressive BBAs (20). The treatment options for BBAs are microsurgery, endovascular therapy (EVT), or a combination of two (4). Comparison of two treatment strategies showed that EVT for BBAs of the ICA is more effective and safe than surgical approaches (5, 16, 21, 22). A systematic review by Rouchaud et al. compared overall EVT techniques and reported that deconstructive techniques have a higher rate of immediate complete angiographic occlusion but a higher risk of ischemic complications when compared to reconstructive techniques (23). A comprehensive review published in 2020 involving 32 studies (684 patients−707 BBAs) compared stent placement, stent-assisted coiling, and flow diversion results for BBAs. According to the review, overall EVT reconstructive techniques indicated a 76.9% long-term complete occlusion rate, 8.9% perioperative complication rate, 76.6% good clinical outcome at final follow-up, and 4.7% mortality rate. In the review, FD subgroup analysis (155 BBAs with and without adjuvant coiling) results for long-term complete occlusion rate, good clinical outcome at final follow-up, and mortality rate were reported to be 75.6, 76.5, and 5.2%, respectively (24). Some studies suggested that FD with adjuvant coiling increases the occlusion rates; however, in their study with a large number of participants, Mokin et al. showed that coiling with FD does not affect the long-term occlusion rates (25–28).

Numerous studies reported good outcome with 68–83% and 63.6–100% complete occlusion rates of ruptured BBAs treated with FD alone (3, 25, 28–32). In this study, good outcome rates at the follow-up (83.3%) and long-term complete occlusion rates (100%) corroborate these earlier results in the literature. Effective results with FD treatment of ruptured BBAs could be attributed to the mechanism of FD devices. Because of the nature of BBAs, treatment not only should focus on the aneurysmal sac but also should reconstruct the parent artery wall. The FD main mechanism is modifying the blood flow in the aneurysmal sac and decreasing the wall shear stress by maintaining laminar flow in the parent artery (6, 24). Despite the aforementioned effective results of flow diversion strategy, concerns regarding incomplete occlusion of aneurysm, the management of antiplatelet therapy, the timing of the procedure, and rebleeding risk in the acute setting of SAH remain. Cagnazzo et al. reviewed the FD treatments in the acute settings of SAH. The BBA subgroup in the review demonstrated a 35% immediate occlusion rate and an 18% overall complication rate (7). Several studies have reported 0–33.3% immediate occlusion rates and 0–27% periprocedural complication rates (25, 33–36). In this study, the immediate occlusion rate and periprocedural complication rate were 16.6%, and these results are consistent with those in the literature.

An unexpected finding by Sceratti et al. based on numerous studies demonstrated that there is a much greater risk of thromboembolism than a significant rebleed effect when using the FD strategy alone in the acute environment of ruptured BBAs (24, 36). In accordance with this finding, this study demonstrated no rebleed or hemorrhagic complication; however, one transient thromboembolic periprocedural complication was observed. It must also be stated that there has been a finding indicating that hemorrhagic complication risk was 6% and rebleed rate was 3%, especially in the first 72 h after SAH (7, 37). A significant percentage of these hemorrhagic complications was recorded as ventriculostomy-related bleedings. EVD was placed in one patient before the treatment with no hemorrhagic complication, and none of the patients required EVD after the treatment in our study. This small size of EVD-required cases is the limitation of the study.

Zhu et al. reported a 13% recurrence rate and 3% procedure-related mortality (37). In our study, no recurrence was observed in a short- or long-term period, and no procedure-related mortality was recorded. Zhu et al. also compared overlapping FDs with a single device strategy and suggested that a single FD may result in a higher rate of good outcome (61.9 vs. 89.9%, respectively) (37). In our study, due to the small number of patients included, this study cannot provide any evidence about overlapping FDs or single device strategy.

Regarding the timing of the treatment, Dossani et al. reported a meta-analysis regarding early vs. delayed flow diversion of ruptured aneurysms (9). Meta-analyses involving 13 articles and 142 patients did not show a difference in overall complication rates between early (2 days or less) and delayed flow diversion and suggested that the timing of flow diversion does not impact the overall complication rate. In the subgroup analyses, the authors demonstrated the safety profile of BBAs. In our study, all patients were treated in the early phase, and results were compatible with relevant data in the literature. Our results suggest that early treatment is safe and effective in the acute setting of SAH. In accordance with delayed treatment of flow diversion in the acute setting of SAH reported by Capocci et al. (five ICA BBAs), similar results for outcomes, complications, and long-term follow-up were also recorded in our study (8). These data must be interpreted with caution because of the small number of patients enrolled and the SAPT regimen used in our study. To our knowledge, this is the first study on FD in BBAs (of the ICA) in the acute setting. In addition to Capocci et al., some other studies suggest treatment deferral because of the high risk associated with DAPT for hemorrhagic complications particularly in cases where EVD placement is necessary (31, 38). Considering the natural progressive course and high risk for rerupture and regrowth of ruptured BBAs, treatment delay is highly debatable and involves high risk. To our knowledge, there is no article focusing on the early treatment of ruptured BBAs treated with FDs. There had been some mixed (early and delayed treatment fashion) data and only one aforementioned delayed treatment safety and efficacy report (3, 8, 25, 30, 33, 35, 38). Our results contribute to the safety profile of FDs in the early treatment period of ruptured BBAs of ICA with a similar outcome and complication rates compared to delayed treatment and overall data regardless of time to treatment. Our sample size is a limitation of the study, and large prospective studies are warranted to validate our findings.

Due to the lack of appropriate platelet inhibition following clopidogrel administration (clopidogrel resistance), which inhibits the P2Y12 adenosine diphosphate (ADP) receptor and has been associated with higher rates of ischemic complications following neuroendovascular procedures (39), our institutional approach is to use new P2Y12 antagonists for intracranial stenting, especially for FDs. Although DAPT with acetylsalicylic acid plus clopidogrel is still the main choice for FDs, Podlasek et al. recently reported in their meta-analyses that new-generation P2Y12 antagonists (ticagrelor and prasugrel) are safe for patients undergoing flow diversion (11). Another study demonstrated the safety of prasugrel loading 50 mg before 2 h from stent delivery for thromboembolic and hemorrhagic complications in ruptured BBAs treated with PED in nine patients (40). In the study, only three patients were treated within 2 days from ictus, and aspirin was used with prasugrel in all patients. There is no consensus about the use and timing of optimal antiplatelet regimen for FDs in the acute setting of SAH yet.

Theoretically, SAPT regimen in the acute setting of SAH is relatively safer than DAPT for hemorrhagic complications and rebleed risk; however, thromboembolic complications may be a concern, especially in the vasospasm period. SAPT use with FD treatment is a new approach, and limited data are available in the literature. In a recent study, ticagrelor was used to prevent thromboembolic complications with Pipeline device (PED) as SAPT (10). Mohammaden et al. demonstrated ticagrelor as a safe and effective antiplatelet in ruptured aneurysms with FDs. In the subgroup analyses, six of 36 aneurysms were BBAs. In their recent study, the authors referred to the studies that reported the effect of acetylsalicylic acid delaying the rate of endothelial cell production and safety of ICA region with a lower chance of device deployment over a perforator (10). The safety and efficacy of acetylsalicylic acid and prasugrel as SAPT for newer FD technologies are reported (41, 42). In this study, we used ticagrelor in one patient and prasugrel in five patients. Accordingly, only one treatment-responsive (vasospasm treated with i.a. nimodipine) thromboembolic complication occurred. To our best knowledge, prasugrel as SAPT for classical FDs used for ruptured BBAs was firstly reported in our study. This study is not generalizable given the lack of a control group, the small number of cases, and the necessary invasive procedures such as EVD. Single-center retrospective analyses of the data are another limitation due to the rarity of the disease. Multicenter large prospective studies are required to confirm findings.

## Conclusion

This single-center experience suggests that early treatment (<2 days) within SAH of ruptured BBAs with FDs is feasible and likely safe, but additional studies with a larger sample are needed to confirm the findings. In the era of new FD technologies with less thrombogenicity, SAPT may be the optimal future antiplatelet regimen for ruptured BBAs. Using ticagrelor and prasugrel as SAPT in the early period of SAH due to BBAs with classical FDs warrants large prospective studies.

## Data Availability Statement

The original contributions presented in the study are included in the article/supplementary material, further inquiries can be directed to the corresponding author.

## Ethics Statement

The studies involving human participants were reviewed and approved by Baskent University Institutional review board. Written informed consent for participation was not required for this study in accordance with the national legislation and the institutional requirements.

## Author Contributions

All authors performed the measurements, planning and supervised the work, drafted the manuscript and designed the figures, discussed the results, and commented on the manuscript equally.

## Conflict of Interest

The authors declare that the research was conducted in the absence of any commercial or financial relationships that could be construed as a potential conflict of interest.

## Publisher's Note

All claims expressed in this article are solely those of the authors and do not necessarily represent those of their affiliated organizations, or those of the publisher, the editors and the reviewers. Any product that may be evaluated in this article, or claim that may be made by its manufacturer, is not guaranteed or endorsed by the publisher.
